# Digital Trauma Load: from Online Videogaming to the new forms of digital social networking

**DOI:** 10.1192/j.eurpsy.2023.143

**Published:** 2023-07-19

**Authors:** L. Orsolini

**Affiliations:** Unit of Clinical Psychiatry, Department of Neurosciences/DIMSC, Polytechnic University of Marche, Ancona, Italy

## Abstract

**Abstract:**

The digital revolution is evolving at an unstoppable pace. The dissemination of interconnected networks, the growth and spread of new digital technology across all ages at different levels, has rapidly led to an increasing usage of the Internet, smartphones and digital social networking, particularly among young people. Smartphones, social networking and digital videogaming platforms are currently being used as primary means of online access, source of information and as preferred mode for social communication and peer interaction. However, despite digital world is progressively replacing the ‘physical world’ concerning socialization process and communication modalities, there is poor attention on the ‘digital trauma load’ experienced by individuals belonging to the Z and alpha generations in term of potential impact of digital instruments on mental health.

**Disclosure of Interest:**

None Declared

